# A Novel Bromodomain Inhibitor Reverses HIV-1 Latency through Specific Binding with BRD4 to Promote Tat and P-TEFb Association

**DOI:** 10.3389/fmicb.2017.01035

**Published:** 2017-06-07

**Authors:** Huachao Huang, Shuai Liu, Maxime Jean, Sydney Simpson, He Huang, Mark Merkley, Tsuyoshi Hayashi, Weili Kong, Irene Rodríguez-Sánchez, Xiaofeng Zhang, Hailemichael O. Yosief, Hongyu Miao, Jianwen Que, James J. Kobie, James Bradner, Netty G. Santoso, Wei Zhang, Jian Zhu

**Affiliations:** ^1^Department of Microbiology and Immunology, University of Rochester Medical CenterRochester, NY, United States; ^2^Department of Chemistry, University of Massachusetts BostonBoston, MA, United States; ^3^Department of Chemistry, Laufer Center for Physical and Quantitative Biology, Stony Brook UniversityStony Brook, NY, United States; ^4^Department of Biostatistics, School of Public Health, University of Texas Health Science CenterHouston, TX, United States; ^5^Department of Medicine, Columbia University Medical CenterNew York, NY, United States; ^6^Department of Medicine, University of Rochester Medical CenterRochester, NY, United States; ^7^Harvard Medical School, Dana-Farber Cancer InstituteBoston, MA, United States

**Keywords:** HIV-1, latency, reactivation, BRD4, LRA, BETi, JQ1, UMB-136

## Abstract

While combinatory antiretroviral therapy (cART) can effectively reduce HIV-1 viremia, it cannot eliminate HIV-1 infection. In the presence of cART, viral reservoirs remain latent, impeding the cure of HIV-1/AIDS. Recently, latency-reversing agents (LRAs) have been developed with the intent of purging latent HIV-1, providing an intriguing strategy for the eradication of the residual viral reservoirs. Our earlier studies show that the first-generation, methyl-triazolo bromodomain, and extra-terminal domain inhibitor (BETi), JQ1, facilitates the reversal of HIV-1 latency. BETis have emerged as a new class of compounds that are promising for this HIV-1 “shock and kill” eradication approach. However, when used as a single drug, JQ1 only modestly reverses HIV-1 latency, which complicates studying the underlining mechanisms. Meanwhile, it has been widely discussed that the induction of latent proviruses is stochastic (Ho et al., 2013). Thus, new BETis are currently under active development with focus on improving potency, ease of synthesis and structural diversity. Using fluorous-tagged multicomponent reactions, we developed a novel second-generation, 3,5-dimethylisoxazole BETi based on an imidazo[1,2-a] pyrazine scaffold, UMB-32. Furthermore, we screened 37 UMB-32 derivatives and identified that one, UMB-136, reactivates HIV-1 in multiple cell models of HIV-1 latency with better efficiency than either JQ1 or UMB-32. UMB-136 enhances HIV-1 transcription and increases viral production through the release of P-TEFb. Importantly, UMB-136 enhances the latency-reversing effects of PKC agonists (prostratin, bryostatin-1) in CD8-depleted PBMCs containing latent viral reservoirs. Our results illustrate that structurally improved BETis, such as UMB-136, may be useful as promising LRAs for HIV-1 eradication.

## Introduction

The discovery of combinatorial antiretroviral therapy (cART) has significantly suppressed human immunodeficiency virus type 1 (HIV-1) infection, and transforming acquired immune deficiency syndrome (AIDS) from an acute lethal disease to a chronic manageable one. However, latent residual virus remains in infected patients on cART, creating the hurdle toward an HIV-1 cure. In recent years a strategy to reverse HIV-1 latency using small molecules has been developed; demonstrating the potential to eliminate the viral reservoirs (Choudhary and Margolis, 2011; Rasmussen et al., 2016). These compounds can initiate viral replication, thereby sensitizing the latent reservoir to (i) virally-mediated cytopathic effects and (ii) host immune attack. Developed latency reversing agents (LRAs) have been tested in both *in vitro* and *ex vivo* HIV-1 latency models. One recently identified class of LRAs is bromodomain and extra-terminal domain inhibitors (BETis) (Banerjee et al., 2012; Bartholomeeusen et al., 2012; Zhu et al., 2012; Boehm et al., 2013; Li et al., 2013). Several studies have convincingly shown that one BETi, JQ1, can reverse HIV-1 latency *ex vivo* with a high efficacy when used in combination with protein kinase C (PKC) agonists (prostratin, bryostatin-1and ingenol-B; Darcis et al., 2015; Laird et al., 2015). However, JQ1 alone shows weak activity in reactivating latent viruses in J-Lat and primary CD4+ T cells (Zhu et al., 2012; Bullen et al., 2014). It also exerts severe cytotoxicity, preventing long term or high dosage treatments. In addition, LRA usage may lead to the provirus becoming insensitive to reactivation, as the HIV-1 latency-reversing effect could be stochastic (Ho et al., 2013). These issues have prompted a need for structurally dissimilar LRAs (ligands as probes of bromodomain function). Other methyl-triazolo BETis are not as potent as JQ1 in reversing HIV-1 latency, possibily due to the fact that they share the similar backbone (Darcis et al., 2015). Collectively these early investigations are informative, but they also indicate that current BETis require improvement for the use as potent LRAs and that other structurally diversified LRAs are needed to be developed.

To increase the structural diversity of chemical ligands for use as probes of bromodomain function, we previously used fluorous-tagged multicomponent reactions to generate a focused chemical library of novel BETis centered on the 3,5-dimethylisoxazole biasing element (McKeown et al., 2014). We explored a variety of chemical scaffolds while keeping the dimethyl isoxazole biasing moiety; and found the para-imidazo[1,2-a] pyridine scaffold to be the most promising lead, and thus subjected it to further studies of structure-activity relationships (SAR). The lead compound using the imidazo [1,2-a] pyrazine scaffold, 32 (UMB-32), demonstrated a strong binding affinity with bromodomain-containing proteins (BRD4 and TAF1) as well as a potent inhibitory effect on BRD4. Additionally, The UMB-32 structure is much easier to synthesize than JQ-1 and allows for the rapid expansion of analogs and modifications. In this study, we evaluated the potency of UMB-32 based BETis as potential new LRAs.

## Methods and materials

### Synthesis of UMB-32 analogs

The syntheses of UMB-32 analogs including UMB-58, -59, -136, and -283 were accomplished using a previously reported two-step synthesis involving a three-component (Groebke–Blackburn–Bienayme) reaction followed by Suzuki coupling (McKeown et al., 2014).

### Computational calculation

AMBER 14 (Case et al., 2005) software was used to implement molecular mechanics Poisson Boltzmann (MM-PBSA; Kollman et al., 2000) and related generalized Born (MM-GBSA; Gohlke et al., 2003), which in turn were used to calculate the relative binding affinities of BETis to BRD4 BD1. The calculated energies were further decomposed into insightful interaction components.

Cartesian coordinates for protein-ligand complexes were determined by aligning second generation BETis (UMB-58, UMG-59, and UMB-136) with the characterized pose of the parental ligand in a crystal structure of UMB-32, bound to BRD4 BD1 (PDB code: 4WIV). The new complexes were acquired and initially minimized using Chimera-1.10.2. MD simulations in explicit solvent [FF14SB force field (Maier et al., 2015), TIP3P solvent model (Jorgensen et al., 1983), 50 ps of heating, 50 ps of density equilibration, 500 ps of constant pressure equilibration at 300 K, followed by 4 production runs of 500 ps long each] were used to obtain the equilibrium conformations for each protein-ligand complex, free protein, and free ligand. Continuum solvents [PB (Maier et al., 2015) and GB-OBC (Onufriev et al., 2004)] replaced explicit solvent in the relative free energy calculation phase. The entropic contribution was not added as it is assumed not to alter the relative binding affinity.

### Cell lines and cell culture

The CD4^+^ T-lymphoid cell lines Jurkat, J-Lat A2 (harboring an LTR-Tat-IRES-GFP construct), J-Lat 6.3, 8.4, 9.2, 10.4 (containing a full-length integrated HIV-1 genome with a defective envelop, that express GFP upon activation). Monocytic THP89GFP cells were kindly provided by David Levy (New York University). Jurkat cells were cultured in RPMI with 10%FBS. THP89GFP and Primary CD4^+^ helper T cells (Sanguine Biosciences and Lonza) were cultured and maintained, respectively, in complete medium (RPMI 1640 with 10% FBS, 1x glutamine, 1x MEM non-essential amino acid solution, and 20 mM HEPES).

### Viruses

Pseudo-typed HIV-1 NL4-3 viruses were produced by using TurboFect (Thermo Scientific) to co-transfect pCG-VSV-G and HIV-1 NL4-3-Luc (ΔEnv) plasmids into HEK293T cells. Full-length HIV-1 NL4-3 (X4 tropic) viruses were created similarly by transfecting pNL4-3 (X4) plasmid into HEK293T cells.

### Regents

JQ1 (SML-1524) was purchased from Sigma-Aldrich. Prostratin (SC-203422), SAHA (SC-220139), and bryostatin-1 (SC-201407) were purchased from Santa Cruz Biotechnology. Antibodies against CD3 (16-0037-85) and CD28 (16-0289-85) were purchased from eBioscience. TGF-β (7754-BH-005), IL-12 (AB-219-NA), and IL-4 (AB-204-NA) antibodies were purchased from R&D System. IL-2 (11011456001) and Nevirapine (4666) were obtained from NIH AIDS Reagent Program.

### Flow cytometry assays

J-Lat A2 cells were cultured in a 48-well plate at 1 × 10^5^ cells per well in a total volume of 200 μl RPMI media supplied with 10% FBS. Wells were treated with 5 uM of compound or DMSO and GFP-positive cells were acquired and analyzed by flow cytometry at 24 h post treatment.

For evaluating the HIV-1 latency-reversing effect of UMB-136 on different reservoirs (lymphocytes and macrophages), J-Lat cell clones (6.3, 8.4, 9.2, and 10.4) and THP89GFP were treated with DMSO, JQ1 (1 μM), or UMB-136 (2.5, 5 μM for J-Lat cells, 5 μM for THP89GFP). For the investigation of synergistic effects JLat clones were treated with UMB-136 (2.5 μM), JQ1 (1 μM), SAHA (0.5 μM) or prostratin (1 μM) or DMSO as a control. Drugs were used alone or in combination. Flow cytometry was conducted at 24 h post treatment and GFP-positive cells were quantified.

All flow cytometry assays were conducted using an Accuri C6 Flow Cytomerer (Accuri, Ann Arbor, MI). Cell debris and aggregates were excluded by gating on the Forward Scatter (FS) and Side Scatter (SS) parameters.

### Pull-down and IP assays

HEK293 cell lysate was incubated with either biotinylated UMB-136 or biotin in DMSO at 4°C overnight with orbital rocking. MyOneTM Streptavidin T1 DynabeadsTM (Invitrogen) were added to the mixture which was then incubation for 2 h. Beads were washed and then boiled to elute precipitated proteins, which were subjected to immunoblotting using BRD4 antibody (Bethyl Laboratories, Inc.) or BRD2 antibody (Santa Cruz).

Tat-Flag expressing HeLa cells were treated with UMB-136 (2.5 μM) or DMSO for 24 h, then incubated with Magnetic Flag Conjugated beads (Clontch) at 4°C for overnight with orbital rocking. Beads were washed and boiled, then immunoblotting was performed using Cyclin T1 antibody (Santa Cruz).

### Cell viability assay

Jurkat cells were cultured in a 48-well plate at 1 × 10^5^ cells per well in a total volume of 250 μl RPMI media with 10% FBS. Compounds (JQ1, UMB-136, or DMSO) were added. After an incubation period of 24 h, cells were lysed. The total ATP content was measured using Cell Titer Glo (Promega).

### Quantitative PCR assay

Effects of the LRAs on HIV transcription were determined by qPCR assay. Jurkat cells were transduced with VSV-G pseudo-typed HIV-1 NL4-3-Luciferase virus for 8 h and treated with compounds (UMB-32, UMB-136, JQ1, or prostratin) for 24 h. Cells were collected and subjected to mRNA extraction (RNeasy® Mini Kit, Qiagen) and reverse transcription (iScript™ cDNA Synthesis Kit, Bio-Rad). The qPCR assays were conducted as previously described with slight modification (Johnston et al., 2009), using the iTaq™ Universal SYBR® Green Supermix (Bio-Rad) on the CFX Connect™ Real-Time PCR System (Bio-Rad). The following qPCR primers were used: Initiation (10–59 bp) (forward, 5′- GTT AGA CCA GAT CTG AGC CT-3′; reverse, 5′-GTG GGT TCC CTA GTT AGC CA-3′); Elongation (29–180 bp) (forward, 5′-TGG GAG CTC TCT GGC TAACT-3′; reverse, 5′-TGC TAG AGA TTT TCC ACA CTG A-3′).

To determine the effect of UMB-136 on HIV-1 replication, Jurkat cells were infected with full-length HIV-1 NL4-3 (X4) viruses for 8 h before being treated with compounds (UMB-136, JQ1, or prostratin) for 24 h. The medium was collected and subjected to viral RNA extraction, reverse transcription, and qPCR assays by using Lenti-XTM qRT-PCR Titration Kit (Clontech) following the manufacturer's instruction.

### Acquisition of primary CD4+ T cells

Primary CD4+ T cells were isolated from either lymphoid tissue (tonsil) or from the peripheral blood of two donors. Tonsils were isolated from HIV-negative donors, with obstructive sleep apnea (OSA), whom required surgical removal of their tonsils. Isolation of tonsillar mononuclear cells (TMC) was performed as previously described (Johnston et al., 2009). In brief, fresh, healthy tonsils kept in ice-cold 1x HBSS with antibiotics (100 U/mL penicillin, 100 ug/mL streptomycin, 5 ug/mL gentamicin, 0.5 ug/mL amphotericin) were cut into 3- to 10-mm fragments with scalpels then pushed through a 3-inch stainless steel sieve using the flat end of a 5-mL plastic syringe plunger. After breaking up any cell clumps, a 35 mL cell suspension was passed through a sterile 40-μM plastic cell strainer (Fisher) and subsequently overlaid on 10-mL Ficoll-Hypque (GE Healthcare), before being centrifuged at 1,000 ×g for 20 min without braking. Mononuclear cells were collected from the interface and washed three times with ice-cold 1x HBSS then re-suspended in cryopreservative medium for storage in liquid nitrogen. Tonsillar CD4+ T cells were purified using the CD4+ T cell Isolation Kit (Miltenyi Biotec) according to the manufacturer's instruction. Peripheral CD4+ T cells were purchased from Lonza.

### Primary CD4+ T cell model of HIV-1 latency

To study the effect LRA effect of UMB-136 we slightly modified a primary CD4+ T cell model of HIV-1 latency, which we had used previously (Bosque and Planelles, 2011; Huang et al., 2015). In brief, primary T cells were activated using CD3/CD28 antibodies and then infected with full-length HIV-1 NL4-3 (X4) viruses. Cells were treated with TGF-β, anti-IL12, and anti-IL4 antibodies (R & D Systems) for non-polarization to allow for the establishment of latent HIV-1. These cells were treated with compounds 2.5 UMB-136 or 1 μM JQ1 day 16 post infection. Twenty-four hours later the compounds were washed away and cells were re-suspended in fresh complete RPMI media. On day 21, 5 days post treatment; the supernatant was collected for quantification of newly produced viruses using the Lent-X™ qRT-PCR Titration Kit (Clontech) following the manufacturer's instruction.

### Isolation of CD8-depleted PBMCs

PBMCs were isolated from fresh whole blood of cART-treated HIV- infected aviremic patients using the Ficoll-Hypaque gradient method, as described previously (Reuse et al., 2009; Bouchat et al., 2012). Whole blood was diluted in 1x PBS (without Ca^2+^ or Mg^2+^) at 1:1 ratio. Then, 26 mL of the cell suspension was gently overlaid onto 14 ml of Ficoll-Paque (GE Healthcare) in a 50 mL conical tube. This was centrifuged for 20 min at 966 xg without braking, at room temperature. The PBMC band from interface was collected and transferred to a new sterile 50 mL conical tube. PBMCs were washed three times using 1x PBS with 5 mM EDTA. Cells were allowed to rest in complete RPMI media in the presence of Nevirapine (600 nM) and IL-2 (30 IU/ml) for 3 days. CD8+ T cells were depleted by negative selection using CD8 MicroBeads (Miltenyi Biotec), following the manufacturer's instructions.

### Nested qPCR assay to quantify HIV-1 reactivation from aviremic samples

We used an ultra-sensitive nested qPCR assay to quantify reactivated HIV-1 viruses according to a previously reported protocol (Mousseau et al., 2015). CD8-depleted PBMCs were cultured at 2 × 10^6^ cells/ml and stimulated with indicated compound combinations for 48 h: UMB-136 (2.5 μM), JQ1 (1 μM), prostration (250 nM), bryostatin-1 (10 nM), anti-CD3/CD28 antibodies (1:1), or DMSO. Supernatants were collected and viral RNA extraction was performed using the QIAmp® Viral RNA kit (Qiagen) following the manufacturer's protocol. Extracted viral RNA was subsequently treated with DNase I (Invitrogen) for 10 min at 25°C then inactivated by EDTA treatment for 10 min at 65°C. A Reverse transcription coupled qPCR assay was carried out using the Superscript® III One-Step RT-PCR System with Platinum® Taq High Fidelity (Life Technologies) in a total volume of 50 μl. Gag gene-specific primers were used for PCR are as follows: forward (Q1), 5′-ATG CCA CGT AAG CGA AAC TCT GGG TCT CTC TGG TTA GAC-3′; reverse (Q2), 5′-CCA TCT CTC TCC TTC TAG C-3′. The following thermal cycles were used: 50°C for 30 min, 94°C for 2 min, and 16 cycles of [94°C for 15 s, 62°C for 30 s, 68°C for 60 s], with a final elongation step at 65°C for 5 min. The pre-amplified RT-PCR products were purified using the QIAquick® PCR Purification Kit (Qiagen) and further subjected to a second nested qPCR assay using the TaqMan® Universal PCR Master Mix (Applied Biosystems) in the total volume of 25 μl. Primers and probes used for nested PCR are as indicated: forward (Q3), 5′-ATG CCA CGT AAG CGA AAC T-3′; reverse (Q4), 5′-CTG AGG GAT CTC TAG TTA CC-3′; probe, 5′-6FAM/CAC TCA AGG CAA GCT TTA TTG AGG C/6-TAMSp-3′. The thermal cycles included one for UNG incubation (50°C for 2 min), followed by initial denaturation (95°C, 10 min), 45 cycles of amplification (95°C, 15 s; 50°C, 20 s; 60°C, 1 min). All PCR assays were conducted on the CFX connect™ real time PCR detection system (Bio-Rad). A serial dilution of HIV-1 IIIB viruses with known concentrations at a series of dilutions were used to create a standard curve for the absolute quantification of reactivated HIV-1 viruses in supernatants.

### Study subjects

cART-treated, HIV-infected, aviremic patients were recruited by the AIDS clinic at the Strong Memorial Hospital of the University of Rochester Medical Center (Rochester, New York). All study subjects had been treated with cART for >3 years, had an undetectable plasma HIV-RNA level (<50 copies/ml) for at least 6 months, and a normal CD4+ T lymphocyte count (>300 cells/mm^3^) at the time of the leukapheresis process. Written informed consent from the study subject was obtained before the enrollment. This study was approved by the University of Rochester Research Subjects Review Board with an assigned number (#RSRB00053667).

## Results

### UMB-136 is a leading UMB-32 based BETi

**Figure 1:**
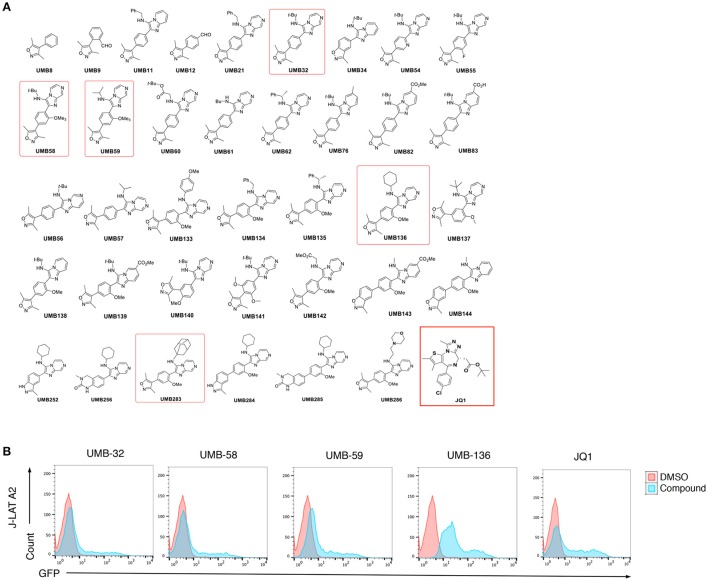
UMB-136 is a leading UMB-32 based BETi. **(B)** J-Lat A2 cells were treated with UMB-32, 58, 59, or 136 (5 μM), or JQ1 (1 μM). GFP-positive cells were acquired and analyzed by flow cytometry. The HIV-reactivated cell population was indicated. **(A)** Chemical structures of 37 UMB-32 analogs. The structures of UMB-32, 58, 59, 136, and JQ1 were highlighted in red frames.

We determined the HIV-1 latency-reversing potential of 37 UMB-32 analogs using J-Lat A2 cells (Figure 1A). Four compounds (UMB-32, UMB-58, UMB-59, and UMB-136) significantly induced HIV-1 reactivation; with UMB-58, UMB-59, and UMB-136 proving to be more potent than UMB-32 and UMB-136 being the most effective of all (Figure 1B). We postulate that this is due to the presence of an additional methoxy group (R-OCH_3_), which is shared by the three aforementioned compounds but not with UMB-32 (Figure 1A). A structural mimic of UMB-136, UMB-283, shows similar potency in reversing HIV-1 latency (Figure S1A). Cell viability assays were performed in Jurkat cells as shown in Figure S1B. 2.5 μM of UMB-136 causes the same viability to 1 μM of JQ1. Thus, for the following experiments, the above concentrations were used for each drug.

### UMB-136 enhances HIV-1 transcription and viral production by releasing P-TEFb

**Figure 2:**
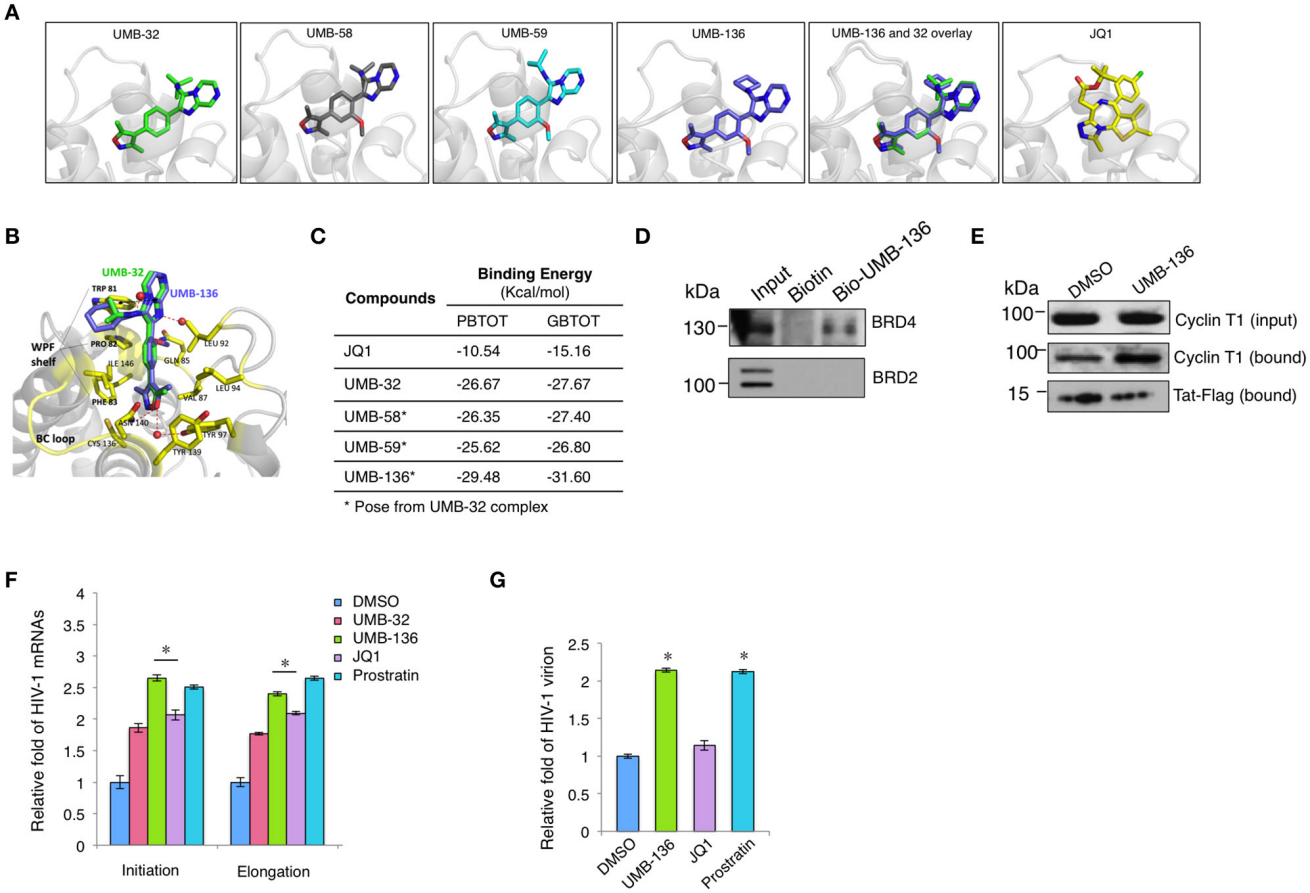
UMB-136 promotes HIV-1replication by releasing P-TEFb through binding to BRD4. **(A)** The predicted binding of UMB-58, 59, and 136 with the BRD4 bromodomain (BD1) according to the available data for UMB-32. **(B)** The superimposition of UMB-136 (violet) onto UMB-32 (green) in complex of BRD4 BD1 (PDB ID: 4WIV; protein: residues in contact with ligand shown in yellow, the rest in gray); hydrogen bonding with crystal waters (red spheres) in dashed red lines, pi-stacking in black dashed line. Graphic representations of **(A,B)** are generated in PyMol Software. **(C)** Calculation of the binding energy (PBTOT—relative total binding energy using MM-PBSA; GBTOT—relative total binding energy using MM-GBSA) for UMB-32, 58, 59, 136, and JQ1. **(D)** Pull-down assay for UMB-136. The lysate of HEK293 cells was incubated with biotinylated UMB-136 or biotin in DMSO at 4°C for overnight with orbital rocking. MyOne™ Streptavidin T1 Dynabeads™ (Invitrogen) were added to the mixture for incubation for 2 h. Beads were washed and boiled to elute the precipitated proteins and subjected to immunoblotting using BRD4 or BRD2 antibody. **(E)** UMB-136 promotes the association of Tat and P-TEFb. Hela cells stably expressing Tat-Flag were treated with UMB-136 (2.5 μM) or DMSO for 24 h, followed by the incubation with Magnetic Flag Conjugated beads (Clontch) at 4°C for overnight. Beads were washed and boiled to elute the precipitated proteins, which were subjected to immunoblotting using Cyclin T1 antibody. **(D,E)** were repeated twice individually. **(F)** Jurkat cells were infected with VSV-G pseudo-typed HIV-1 NL4-3 virus, and treated with UMB-32 (2.5 μM), UMB-136 (2.5 μM), JQ1 (1 μM), prostratin (1 μM), or DMSO. The level of viral transcripts at different length was determined by RT-qPCR using the indicated primers. **(G)** Jurkat cells were infected with full length NL4-3 (X4 tropic), and treated with UMB-136 (2.5 μM), JQ1 (1 μM), or prostratin (1 μM). The newly produced viruses in the supernatant were measured by qPCR. Results were presented as mean ± s.e.m. of two or three biological replicates ^*^p < 0.05, *t-*test.

Structurally, out of all UMB-32 derivatives UMB-136 exclusively contains a cyclohexane group (Figure 1A). We questioned whether the binding of UMB-32 analogs with bromodomains (BDs) of BRD4 may correlate with their potency to reverse HIV-1 latency. We calculated the binding energies of UMB-58, UMB-59, and UMB-136 with BRD4 BD1, by imposing these compounds onto available structure data of UMB-32 bound to BRD4 BD1(McKeown et al., 2014; Figures 2A,B). Notably, UMB-136 had the lowest binding energy (PBTOT: −29.48 Kcal/mol; GBTOT: −31.60 Kcal/mol) among all UMB-32 analogs and was also lower than that of JQ1 (Figure 2C). This is consistent with our previous HIV-1 latency reversing assay results where UMB-136 was the most effective of compared compounds. In addition to other groups we have previously shown that BRD4 inhibition by JQ1 increases Tat-dependent transcriptional elongation and Tat-P-TEFb association (Jang et al., 2005; Yang et al., 2005; Zhu et al., 2012). To molecularly dissect UMB-136's effect on HIV-1 transcription, we performed a protein pull-down assay utilizing biotiny-lated UMB-136. Our results showed that UMB-136 binds to endogenous BRD4 but not BRD2 (Figure 2D). Furthermore, UMB-136 treatment resulted in greater protein association between P-TEFb (CCNT1) and Tat (Figure 2E).

As BETis have been shown to increase HIV-1 transcription we wanted to determine the effect of UMB-136 on HIV-1 transcription. We tested this in VSV-G pseudo-typed HIV-1 NL4-3-Luciferase virus infected Jurkat cells. By using qPCR assays to measure HIV-1 transcript length, we found that UMB-136 enhances both transcriptional initiation and elongation of HIV-1 in, Jurkat cells, similar to prostratin (Figure 2F). We noticed that although UMB-136 acts significantly better than JQ1 in inducing transcription that the effect is not dramatic. This may be due to that the single round of infection of pseudo-type virus. Thus, we repeated the experiment on full replication competent NL4-3 infected Jurkat cells in order to determine the effect of UMB-136 with respect to full replication. UMB-136 enhanced viral production in this full-length viral model, comparable to prostratin, while JQ1 did not (Figure 2G). This is consistent with earlier studies, which showed that JQ1 alone does not enhance HIV-1 production in primary cell models (Zhu et al., 2012; Bullen et al., 2014).

### UMB-136 reverses HIV-1 latency in multiple cell models of HIV-1 latency

**Figure 3:**
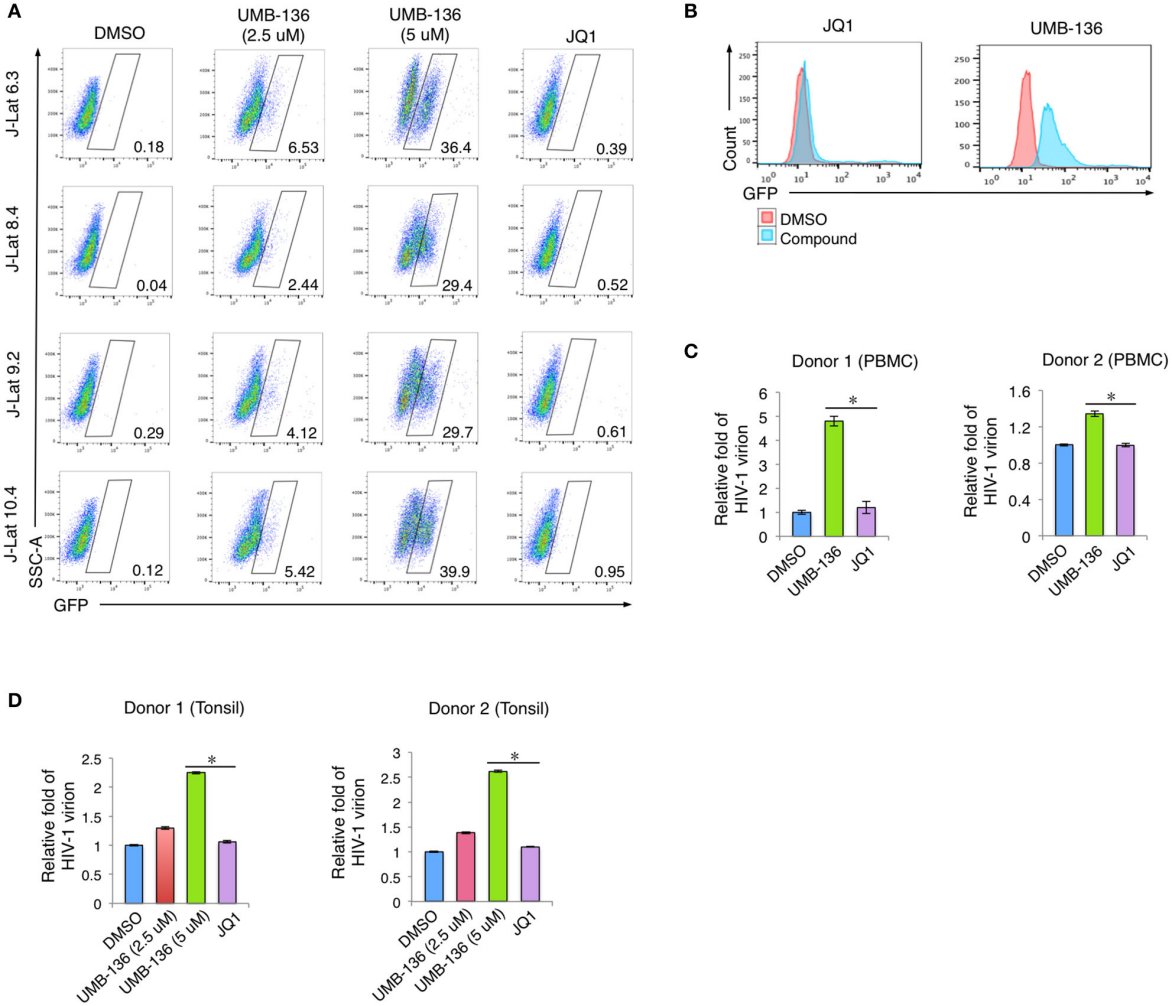
UMB-136 reverses HIV-1 latency from multiple cell models of HIV-1 latency. **(A)** Multiple J-Lat cell clones (6.3, 8.4, 9.2, and 10.4, CD4+ T cell background) were treated with UMB-136 (2.5 or 5 μM) or JQ1 (1 μM). GFP-positive cells were acquired and analyzed by flow cytometry. Percentage of GFP-positive cells was determined. One representative of three independent experiments was shown. **(B)** THP89GFP cells (monocyte/macrophage background) were treated with UMB-136 (5 μM) or JQ1 (1 μM). GFP-positive cells were acquired and analyzed by flow cytometry. The HIV-reactivated cell population was indicated. **(C)** HIV-1 latency was established in the CD4+ T cells isolated from the peripheral blood mononuclear cells (PBMCs) of 2 donors, following a previously reported primary cell model (Vicente Planelles). Cells latently infected with wild-type HIV-1 NL4-3 viruses were treated with UMB-136 (2.5 μM) or JQ1 (1 μM). The viral mRNAs from newly produced HIV-1 viruses in supernatant were measured by qRT-PCR assay. **(D)** The same assay in **(D)** was conducted using the CD4+ T cells isolated from the tonsillar mononuclear cells (TMCs) of 2 donors. Results were presented as mean ± s.e.m. (*n* = *3*); ^*^p < 0.05, *t*-test.

To fully determine the potential of UMB-136 in reactivating full-length HIV-1 genome in multiple cell models of HIV-1 latency, flow cytometry was performed on several J-Lat full-length (dEnv) clones (6.3, 8.4, 9.2, and 10.4) treated with indicated LRAs in Figure 3A. UMB-136 treatment dramatically reversed HIV-1 latency at both low (2.5 μM) and high (5 μM) doses, while, consistent with our previous studies, JQ1 elicited no observable effect (Zhu et al., 2012). We also compared UMB-136 and JQ1 in THP89GFP cells, a previously described HIV-1 latency monocytic cell line containing a wild-type HIV-1 89.6 strain engineered to express GFP (Kutsch et al., 2002). UMB-136 treatment leads to substantial reactivation of where JQ1 did not (Figure 3B). To further evaluate the effect of UMB-136, we used a primary CD4+ T cell model of HIV-1 latency originally developed by Planelles' group (Bliss, 1939; Bosque and Planelles, 2009, 2011; Huang et al., 2015) as described in Section Methods and Materials. Naïve CD4+ T cells were isolated from peripheral blood mononuclear cells (PBMCs) or tonsillar mononuclear cells (TMCs) from two donors. Latency-reversing effects were determined by measuring the luciferase activity, which showed that UMB-136 efficiently reactivated latent virus in non-polarized, memory CD4+ T cells isolated from either blood or lymph node (tonsil) (Figures 3C,D). We also determined that UMB-136 does not induce cytokine release in primary CD4+ T cells from healthy donor (Figure S2).

### UMB-136 synergizes with other LRAs to reverse HIV-1 latency in Jurkat-based latency models

**Figure 4:**
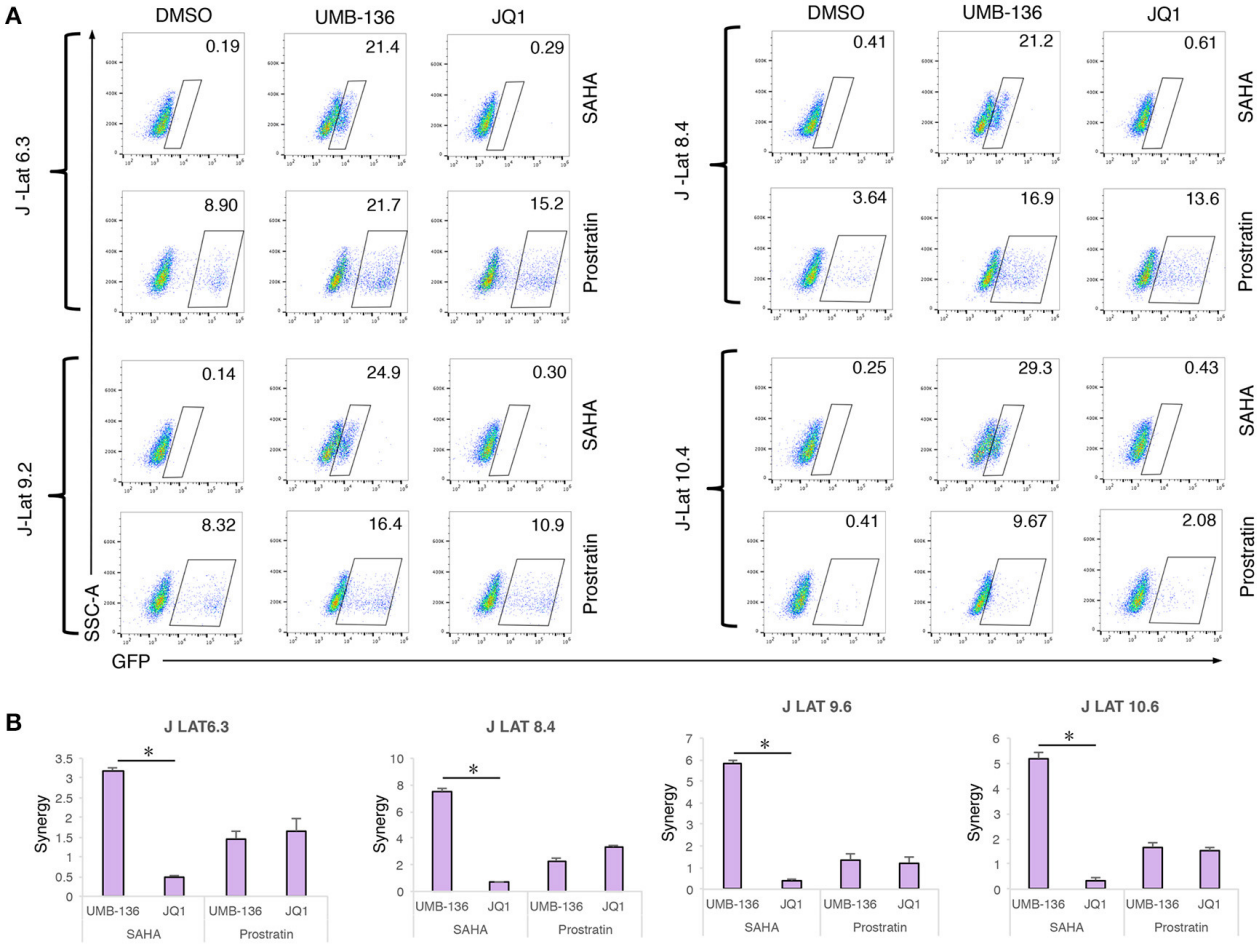
UMB-136 synergizes with other LRAs to reverse HIV-1 latency in J-Lat cell lines. **(A)** Multiple J-Lat cell clones (6.3, 8.4, 9.2, and 10.4) were treated with UMB-136 (2.5 μM) or JQ1 (1 μM) in combination with an HDACi (SAHA, 0.5 μM) or a PKC agonist (prostratin, 1 μM). GFP-positive cells were acquired and analyzed by flow cytometry. Percentage of GFP-positive cells was measured. One representative of three independent experiments was shown. **(B)** The synergistic analysis of LRA combinations. The synergy of LRA combinations in Figure 3A was evaluated using the Bliss independence model (Bliss, 1939). Data are represented as the mean ± *SD* of biological duplicates. ^*^p < 0.05, *t*-test.

Recent studies show that a combination of BETis (JQ1) with other types of LRAs, specifically PKC agonists (prostratin, ingenol-B, and bryostatin-1), is one of the most effective combinations to reactivate latent HIV-1 in reservoir cells (Darcis et al., 2015; Laird et al., 2015). It is even comparable to stimulation using anti-CD3/CD28 antibodies in some cases. Since UMB-136 when used as a single LRA showed better effect than JQ1 (Figure 3), we next wanted to determine whether UMB-136 would also exceed JQ1 when used in LRA combinations. We co-treated multiple J-Lat clones (6.3, 8.4, 9.2, and 10.4) with UMB-136 (2.5 μM) or JQ1 in combination with vorinostat/SAHA, a histone deacetylation inhibitor (HDACi), or prostratin. In both combinations, UMB-136 had a greater effect of HIV-1 reactivation than its JQ1 counterpart (Figure 4A). UMB-136+SAHA and UMB-136+prostratin both yield a statistically significant synergistic effect in reversing HIV-1, while JQ1 only significantly synergizes with prostratin based on the Bliss independence model (Bliss, 1939; Figure 4B).

### Combined treatment with UMB-136 and PKC activators reverses HIV-1 latency in patient-derived resting CD4+ T cells

**Figure 5:**
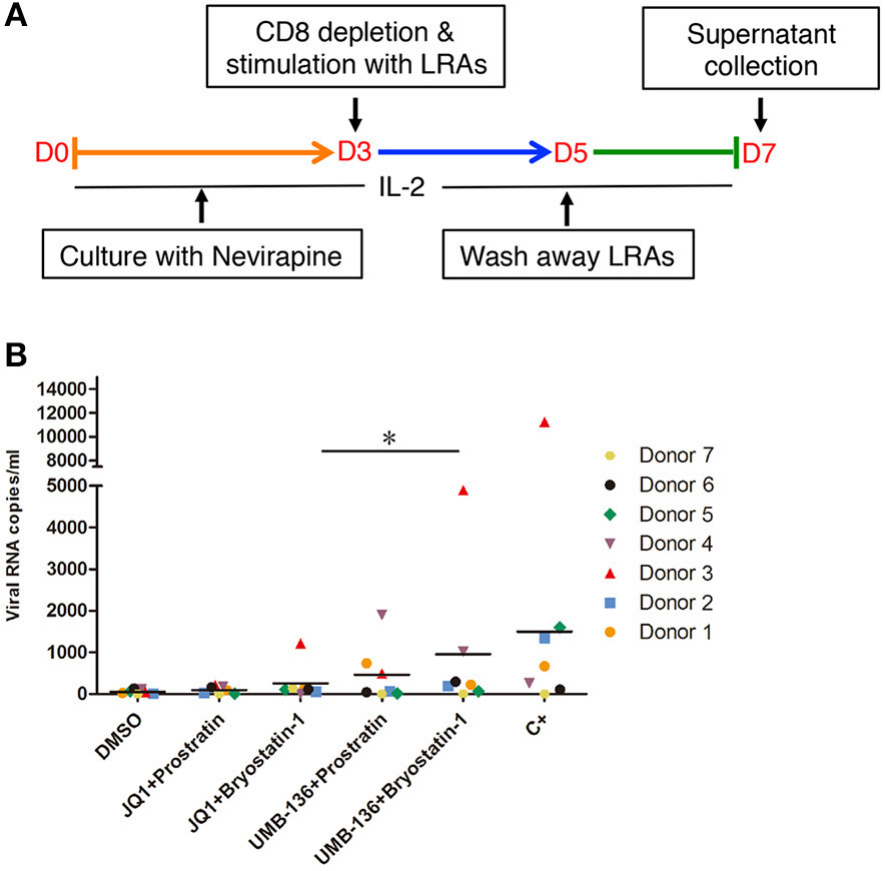
Combined treatment with UMB-136 and Bryostatin-1 reverses HIV-1 latency in patient-derived CD8-depleted PBMCs. **(A,B)** CD8-depleted PBMCs isolated from seven cART-treated, HIV-positive aviremic patients were stimulated with UMB-136 (2.5 μM), or JQ1 (1 μM) in combination with PKC agonists (prostratin, 250 nM; bryostatin-1, 10 nM) to reactivate latent HIV-1 from reservoir cells. The viral transcripts from newly produced HIV-1 viruses in the supernatant were measured by a quantitative nested RT-qPCR assay. C+: antibodies against CD3 and CD28. ^*^P < 0.05, wilcoxon signed rank test.

We further evaluated the LRA combinations of BETis (JQ1, UMB-136) and PKC agonists (prostratin, bryostatin-1) *ex vivo* using CD8-depleted PBMCs isolated from multiple aviremic HIV-infected patients with an undetectable viral load (<50 copies/ml) and normal CD4 count (>300 cells/mm^3^). We saw that a combination of UMB-136 with PKC agonists (prostratin or bryostatin-1) reversed HIV-1 latency (Figures 5A,B). Importantly, while JQ1 when used in combination with bryostatin-1 has been reported to potently reverse HIV-1 latency (Darcis et al., 2015; Laird et al., 2015); UMB-136 + bryostatin-1 notably elicited an even greater viral reactivation. To consider drug toxicity we measured cell death due to LRA treatments and found that UMB-136 and prostratin/bryostatin-1 combinations generally result in less cytotoxicity than JQ1 and prostratin/bryostatin-1 combinations (Figure S2B).

## Discussion

BET proteins are epigenetic readers that regulate transcription through binding of acetylated histones. A series of BETis have been recently developed as promising anticancer drugs. JQ1 is the first-generation, methyl-triazolo based BETi with a nanomolar affinity for BET proteins (BRD2, BRD3, BRD4, and BRDT; Filippakopoulos et al., 2010). The discovery of JQ1 has resulted in the extensive investigation of its therapeutic potential to treat malignancies (Filippakopoulos et al., 2010; Delmore et al., 2011; Zuber et al., 2011), inflammation (Belkina et al., 2013; Chen et al., 2016), and viral infections (Palermo et al., 2011; Wang et al., 2012). Several other methyl-triazolo BETis (iBET, iBET-151, PFI-1) have also been developed and advanced to human clinical investigation (ClinicalTrials.gov). In terms of the application as LRAs, JQ1 has also proved to be the best among these first-generation BETis (Darcis et al., 2015). However, a major concern is that when used as a single LRA, JQ1 fails to efficiently reactivate the latently infected HIV-1 proviruses from most latency cell lines as well as in primary CD4+ T cells (Zhu et al., 2012). This could be due to that, the latently infected HIV-1 proviruses are insensitive to JQ1 induction or that the HIV-1 latency-reversing effects of LRAs (JQ1) are stochastic which has been widely discussed in the field (Ho et al., 2013). Use of structurally diversified BETis may solve this issue. However, while there are at least a dozen of HDACis and PKC agonists that can be selected for the “shock and kill” approach, so far JQ1 is the only BETi to be actively investigated in recent years. In order to form a personalized regimen for HIV-1 eradication, structurally diversified BETis are needed. These concerns have prompted us to develop novel BETis which are structurally distinct from JQ1 for LRA application. Using a 3,5-dimethylisoxazole moiety we recently developed UMB-32, a second-generation BETi which possesses a distinct imidazo[1,2-a]pyrazine scaffold (McKeown et al., 2014). This facile synthetic strategy and biochemical platform allow the efficient optimization of UMB-32 BETi analogs.

We screened a small set of UMB-32 BETi analogs measuring their HIV-1 latency-reversing potential and identified UMB-136 as the best candidate (Figure 1B). The *N*-cyclohexyl group at position 3 of the imidazo[1,2-a] pyrazine in conjunction with the methoxy group position 2 of the benzene ring appear important in improving the potency of the parental UMB-32 (Figure 1A). This hypothesis is supported by the predicted binding of UMB-136 to BRD4 BD1 with lower energy than UMB-32, assuming that UMB-136 occupies the active site of BRD4 BD1 with a similar binding pose as compared to UMB-32 (Figure 2A). This allows conservation of all previously described interactions including: hydrogen bonding to surrounding asparagine and structural waters, and pi-stacking with tryptophan. Furthermore, the addition of cyclohexane and methoxy groups both enhance the binding; with the larger sized cyclohexane group replacing the t-Bu in UMB-32, and burying itself even deeper into the groove between the WPF shelf and the BC loop. And the methoxy group, which is connected to the central phenyl ring, is very likely to bring in additional complementary integration or even hydrophobic interactions (Figure 2B). These postulates are consistent with performed binding energy calculations (Figure 2C). UMB-283, which significantly mimics the chemical structure of UMB-136, maintains a similar potency, reiterating the beneficial effect of the cyclohexane and methoxy groups (Figure S1A).

Although, most studies of BETis focus on the evaluation of their anti-cancer therapeutic potentials, recent reports illustrate that BETis as a new class of HIV-1 LRAs to facilitate the eradication of HIV-1 latent reservoirs (Karn, 2013; Rice, 2013). This observation may be due to the BETis driven release of sequestered P-TEFb and promotion of RNA pol-II activity at the HIV-1 LTR (Archin et al., 2014), which we confirmed for both JQ1 and UMB-136 (Figure 2E). A thorough comparison showed that among first generation of BETis (JQ1, I-BET, I-BET151) tested to reverse HIV-1 latency, that when combined with a PKC agonist (bryostatin-1) that JQ1 is the most effective. However, JQ1 alone is inefficient to reverse viral latency in primary CD4+ T cell models or cART-treated HIV-1 aviremic patient's CD8 depleted PBMCs (Zhu et al., 2012; Darcis et al., 2015). Our results show that UMB-136 alone is capable of reversing latent HIV-1 in several J-Lat cell clones (Figure 3A) as well as in monocytes harboring latent proviruses (Figure 3B). Similarly, UMB-136 alone enables the reversal of HIV-1 latency in a primary CD4+ T cell model, while JQ1 fails to do so (Figures 3C,D). Protein pull-down assays further support that UMB-136 induces reversal of viral latency through specific binding with BRD4 (Figure 2D). However, an earlier report suggests that both BRD4, BRD2 are expected protein targets of JQ1 in reactivating latent HIV-1 (Boehm et al., 2013). It is a possibility that there may be some redundancy between BRD factors in regulating HIV-1 transcription; but, given that the interaction of BRD4 and P-TEFb is well characterized we postulate that BRD4 is the main player among BRD factors or that at least that they may affect HIV-1 transcription through P-TEFb. In terms of LRA combination, UMB-136 also leads to a greater effect of HIV-1 reactivation than JQ1, producing a synergistic effect when combined with HDACis (vorinostat/SAHA) or PKC agonists (prostratin) while JQ1+SAHA combination minimally reactivates latent HIV-1 in J-Lat cell clones (Figure 4A). Similar results are observed in CD8-depleted PBMCs from cART-treated HIV-1 aviremic patients when cells are treated with either drug in combination with PKC agonists (bryostatin-1, prostratin; Figure 5B). However, we must also consider the diverse nature of CD8-depleted PBMCS with respect to cell type. Determination of the location of initial provirus production due to BETi induction would improve our understanding of drug mechanisms when drugs of this class are used as LRAs.

HIV-1 latency-reversing assays across multiple latency cell models, including a set of J-Lat clones, monocytes (THP89GFP), primary CD4+ T cells (Figures 3, 4), demonstrate that UMB-136, whether alone or combined with other LRAs, is more potent than JQ1 in reactivating latent HIV-1. Meanwhile, we observe that across different cell line models and *ex vivo* models, the effect of UMB-136 varies although it shows significant improvement when compared to JQ1. An in-depth comparison of latent HIV-1 reactivation in multiple cell model systems and resting CD4+ T cells from aviremic patients performed by Planelles' group (Spina et al., 2013) found that most cells models demonstrate skewed sensitivities toward or against specific drug classes used in HIV reactivation. As HIV-1 latency cell lines are usually established using the cancer cells, in which cellular metabolism and signaling would be quite different from primary cells this is not too surprising. However, even in primary CD4+ T cell models of HIV- latency, response to the same set of LRAs can be quite varied between models. This may be due to the different approaches used to establish HIV-1 latency in these models. However, importantly, co-treatment with bryostatin-1 shows statistical significance between UMB-136 and JQ1 in aviremic samples (Figure 5B). Earlier studies from two individual groups concluded that the combination of JQ1 with bryostatin-1 is one of the best LRA pairs to reverse HIV-1 latency, comparable to the positive control simulation (CD3/CD28 antibodies) (Darcis et al., 2015; Laird et al., 2015). Use of UMB-136 for such a therapy would lead to a better efficiency than JQ1. Our study also demonstrates the use of the HIV-1 latency-reversing assay as quick and convenient cell-based assay to evaluate the potency of new BETis. As this assay is independent of cell proliferation and growth assays, both of which are routinely used to test BETi-treated cancer cells, we believe that it would be a valuable cross validation of drug effect for new BETis. We have used this assay to successfully identify a leading compound, UMB-136, from a set of UMB-32 analogs. Due our synthetic approach's simplicity (McKeown et al., 2014), we expect that more profound investigation will be initiated to dissect the structure-activity relationship (SAR) of UMB-136. A larger set of UMB-136 analogs will be synthesized for testing using the HIV-1 latency-reversing assay, as improved BETis would be valuable for the application in both anti-tumor and anti-HIV therapies.

## Author contributions

HH, WZ, and JZ conceived and designed the experiments. HH conducted most of the experiments, analyzed the results. SL, MJ, SS, HeH, IR, and HM conducted some of the experiments. MM provided the tonsil tissues. HH and JZ wrote the manuscript. All the authors reviewed the manuscript.

### Conflict of interest statement

The authors declare that the research was conducted in the absence of any commercial or financial relationships that could be construed as a potential conflict of interest.

## Supplementary material

Figure S1 **(A)** The HIV-1 latency-reversing potential of a structural mimic of UMB-136, UMB-283. J-Lat A2 cells were treated with UMB-136, UMB-283, or DMSO at indicated concentrations for 24 h and followed by flow cytometry to sort GFP positive cells. **(B)** Cytotoxicity of UMB-136 and JQ1. Jurkat cells were treated with drugs at a series of concentrations as indicated for 24 h and subjected to measurement of total ATP levels using Cell Titer Glo (Promega).

Figure S2 **(A)** The effect of UMB-136 on the cytokine release of primary CD4+ T cells. Peripheral CD4+ T cells were treated with JQ1, UMB-136, or DMSO. Cells were then treated with anti-CD3 and CD28 antibodies. The indicated cytokines were measured using the Multi-Analyte ELISArray (QIAGEN) following the manufacturer's instruction. **(B)** Live/dead cell assay of the aviremic samples treated with LRAs. CD8-depleted PBMCs from four aviremic donors were processed as shown in Figure 4B. The effect of LRA treatment on cell death was determined using LIVE/DEADTM Fixable Green Dead Cell Stain Kit (Invitrogen) according to the manufacturer's instruction.

## Abbreviations

cART: combinatory antiretroviral therapy
BETi: bromodomain and extra-terminal domain inhibitor
LTR: long terminal repeat
BRD4: bromodomain-containing protein 4
SAR: structure-activity relationship
PBMC: Peripheral blood mononuclear cell.
