# Supplementary figures and images for: A Novel Bromodomain Inhibitor Reverses HIV-1 Latency through Specific Binding with BRD4 to Promote Tat and P-TEFb Association
Source: Front Microbiol. 2017 Jun 7;8:1035. doi: 10.3389/fmicb.2017.01035 (PMC5461361; doi:10.3389/fmicb.2017.01035)

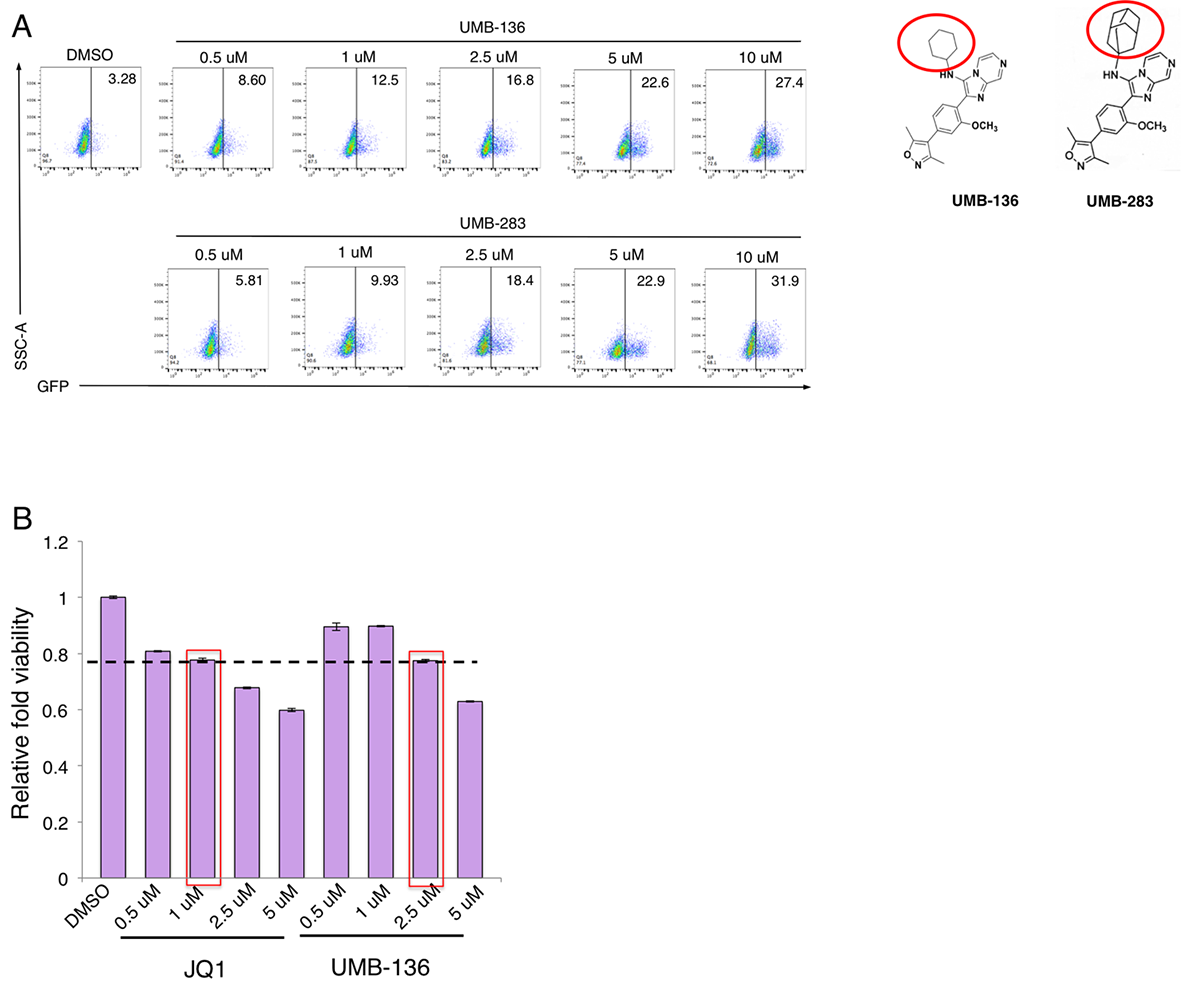

Supplement: Figure S1 — (A) The HIV-1 latency-reversing potential of a structural mimic of UMB-136, UMB-283. J-Lat A2 cells were treated with UMB-136, UMB-283, or DMSO at indicated concentrations for 24 h and followed by flow cytometry to sort GFP positive cells. (B) Cytotoxicity of UMB-136 and JQ1. Jurkat cells were treated with drugs at a series of concentrations as indicated for 24 h and subjected to measurement of total ATP levels using Cell Titer Glo (Promega). [file Image1.tif]

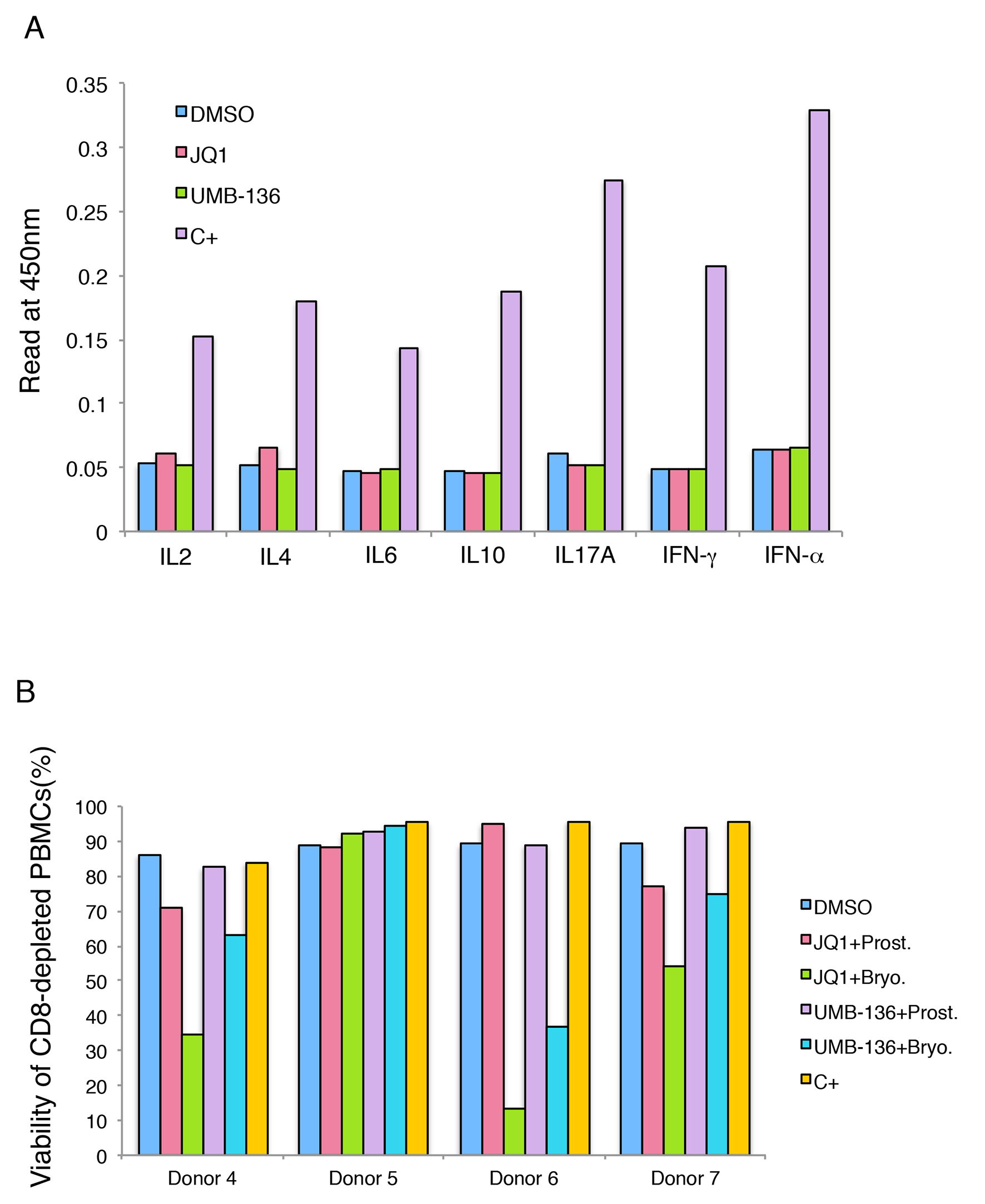

Supplement: Figure S2 — (A) The effect of UMB-136 on the cytokine release of primary CD4+ T cells. Peripheral CD4+ T cells were treated with JQ1, UMB-136, or DMSO. Cells were then treated with anti-CD3 and CD28 antibodies. The indicated cytokines were measured using the Multi-Analyte ELISArray (QIAGEN) following the manufacturer's instruction. (B) Live/dead cell assay of the aviremic samples treated with LRAs. CD8-depleted PBMCs from four aviremic donors were processed as shown in Figure 4B. The effect of LRA treatment on cell death was determined using LIVE/DEADTM Fixable Green Dead Cell Stain Kit (Invitrogen) according to the manufacturer's instruction. [file Image2.TIF]
